# Development and Preliminary Evaluation of a Questionnaire on Parental Informed Consent in Neonatal Research

**DOI:** 10.3390/children13091227

**Published:** 2026-09-10

**Authors:** Maria Lampridou, Abraham Pouliakis, Vasiliki Mougiou, Eleni Katsianou, Aikaterini Konstantinidi, Martha Theodoraki, Zoi Iliodromiti, Theodora Boutsikou, Nicoletta Iacovidou, Rozeta Sokou

**Affiliations:** 1 Neonatal Intensive Care Unit, “Agios Panteleimon” General Hospital of Nikaia, 184 54 Piraeus, Greece; marialampridou1@hotmail.com (M.L.); kmaronia@gmail.com (A.K.);; 2 2nd Department of Pathology, University General Hospital Attikon, School of Medicine, National and Kapodistrian University of Athens, 124 62 Haidari, Greece; apouliak@med.uoa.gr; 3 Neonatal Department, Aretaieio Hospital, School of Medicine, National and Kapodistrian University of Athens, 115 28 Athens, Greece; vassouli@hotmail.com (V.M.); elenifwkou@gmail.com (E.K.); ziliodromiti@med.uoa.gr (Z.I.); theobtsk@med.uoa.gr (T.B.); niakobid@med.uoa.gr (N.I.)

**Keywords:** neonates, parental consent, neonatal research, questionnaire, decision-making processes, informed consent

## Abstract

**Background:** Advances in neonatology and paediatric pharmacology have markedly improved neonatal survival, particularly among preterm and critically ill infants. At the same time, clinical research constitutes the cornerstone for generation of evidence on the safety and efficacy of therapeutic interventions. However, neonatal participation in research relies on parental informed consent, a complex and multifactorial process shaped by cognitive, emotional, and cultural factors. In Greece, evidence regarding the determinants of parental consent for neonatal clinical research remains scarce. The aim of this study was to develop and conduct a preliminary evaluation of a questionnaire designed to assess parents’ perceptions and attitudes toward informed consent for participation of their offspring in neonatal clinical studies. **Methods:** A structured questionnaire was developed based on the international literature and the principles of informed consent. The instrument comprised five domains assessing knowledge and understanding of research, the informed consent process, trust in healthcare professionals, parental emotions, and sociodemographic characteristics. Content validity was assessed by an expert panel, followed by pilot testing in 20 parents. Preliminary evaluation of temporal stability was performed using Cohen’s kappa coefficient and weighted kappa. The study was conducted in accordance with the principles of the Declaration of Helsinki. **Results:** The questionnaire showed variable test–retest agreement across items and domains in the pilot sample. Domains covering cognitive aspects of research participation exhibited greater stability over time compared with emotional dimensions, which showed higher variability between measurements. Questions related to study type and trust in healthcare professionals demonstrated higher levels of reliability. **Conclusions:** This study describes the development and preliminary evaluation of a questionnaire designed to explore factors potentially influencing parental consent for participation in neonatal research in Greece. The findings provide preliminary evidence regarding the temporal stability of individual questionnaire items while also identifying items that may require further evaluation or refinement. Further evaluation in larger and more diverse populations, including a more comprehensive assessment of its measurement properties, is warranted before broader research application.

## 1. Introduction

Rapid advances in neonatology and paediatric pharmacology have significantly contributed to the improvement of survival and quality of life of newborns, particularly preterm and critically ill infants [1,2]. Despite significant progress in medical science, neonates remain one of the most vulnerable and least studied populations when it comes to pharmacotherapy and participation in clinical research [2]. For many years, children—and especially neonates—were described as “therapeutic orphans,” as a large proportion of medications administered to this population were used without adequate scientific evidence, either off-label or without specific authorization for neonatal use [2,3]. The scarcity of data on drug dosing, pharmacokinetics, safety, and efficacy of medications in neonates emphasizes the urgent need for rigorous clinical research in this population [2,4].

Clinical research represents the cornerstone for evaluation of the safety and efficacy of therapeutic interventions [1]. Conducting research in neonates is particularly important, as they exhibit distinct developmental and physiological characteristics that substantially affect their response to medications, distinguishing them from both adults and older children [2]. Factors such as organ immaturity, distinct pharmacokinetic and pharmacodynamic profiles, and the presence of conditions specific to the neonatal period render the applicability of evidence derived from adult or paediatric studies unsuitable for neonatal populations [4]. Furthermore, the complex healthcare needs of preterm infants and those with very low birth weight (VLBW) necessitate tailored therapeutic approaches and further neonatal research for generation of additional high-quality evidence.

Despite their importance, clinical studies in neonatology are associated with major practical, scientific, and ethical challenges [4]. The limited availability of eligible participants, the heterogeneity of the neonatal population, the rarity of certain conditions, and the difficulty in identifying reliable clinical markers constitute significant obstacles to the design and conduct of clinical trials [1,4]. In addition, the scarcity of neonatal-specific pharmacokinetic and pharmacodynamic data and the lack of validated biomarkers and technological equipment specifically designed for neonatal use further complicate the conduct of research in this population [1,5].

Obtaining parental informed consent represents one of the greatest challenges in neonatal research [5]. Unlike adult patients, neonates are unable to make decisions regarding their participation in clinical trials, and this decision rests with their parents or legal guardians [6]. Parents of infants admitted to neonatal intensive care units (NICUs) often experience considerable stress, emotional distress, and psychological vulnerability, particularly when it comes to preterm or critically ill neonates [2]. The need to make fast and complex decisions, combined with the challenges of understanding medical and research-related information, may substantially affect their ability to provide fully informed and voluntary consent. Furthermore, informed consent procedures vary across countries, as different legal and ethical frameworks determine whether consent needs to be obtained from one or both parents [2,7].

Understanding parental attitudes toward informed consent for participation in clinical research, as well as their willingness or reluctance to engage actively in care-related decision-making, is essential for promoting an anthropocentric and ethical approach to neonatal care [8,9]. Nevertheless, research exploring parents’ perspectives on the participation of their newborns in clinical studies remains limited worldwide. Existing evidence is incomplete and frequently focuses on broader parental populations, with relatively little focus specifically to the neonatal subpopulation. This gap is even more pronounced in Greece, where few studies have systematically examined parental attitudes, perceptions, and factors affecting decision-making regarding neonatal participation in research protocols. The limited availability of validated and culturally adjusted measurement tools highlights the need for the development and systematic evaluation of a questionnaire addressing parental perspectives on neonatal research within the Greek sociocultural context.

The aim of this study was to develop and conduct a preliminary evaluation of a questionnaire designed to assess parental attitudes, perceptions, knowledge, and emotions regarding consent for neonatal participation in clinical research. Specifically, the present study focused on content validity, pilot testing, and test–retest reliability as initial steps in the evaluation of the instrument.

## 2. Materials and Methods

### 2.1. Description of the Tool

This study employed a methodological research design aimed at the development and preliminary evaluation of a questionnaire entitled *“Parental Attitudes and Perceptions Regarding Consent for Neonatal Participation in Clinical Research in Greece.”* The instrument was designed to assess parents’ attitudes, perceptions, knowledge, and emotional responses concerning their consent for the participation of their newborn babies in clinical studies. The questionnaire was conceived as a descriptive multidomain instrument, in which items are interpreted individually within their respective thematic domains rather than as indicators of a single underlying latent construct. Accordingly, it was not designed to generate an overall total score or a single composite measure of parental consent. The development and validation of the questionnaire followed sequential steps that included: (a) a comprehensive literature review and item generation; (b) development of the preliminary version of the questionnaire and assessment of content validity by an expert panel; (c) pilot testing of the instrument in a representative sample of parents; and (d) evaluation of the questionnaire’s clarity and test–retest reliability.

### 2.2. Development of the Questionnaire

The development of the questionnaire was based on an extensive review of the international literature on parental consent for neonatal participation in clinical research, the informed consent process, parental decision-making in the context of neonatal hospitalization, the principles of family-centred care, and ethical issues related to neonatal research. The development of questions was also guided by the principles of the Declaration of Helsinki, the Oviedo Convention, and the relevant European and national regulatory frameworks governing biomedical research involving minors, as well as international initiatives and regulatory frameworks, including the European Paediatric Regulation, the European Network of Paediatric Research at the European Medicines Agency (Enpr-EMA), and other international neonatal research networks aiming to carry out and facilitate safe, ethically sound, and scientifically rigorous clinical research in neonates [10,11].

The initial pool of questions was developed using a combination of theoretical and empirical evidence. A systematic review of the available literature was conducted to identify factors associated with parental consent and decision-making regarding neonatal research participation [2,4,7,12,13]. As part of the questionnaire development process, previously published studies investigating parental attitudes toward the participation of children in clinical research were reviewed, including studies employing questionnaires in different countries and settings [8,9]. These studies informed the identification of relevant themes and areas to be addressed; however, the present questionnaire was developed specifically for the Greek neonatal context rather than being a direct translation or cross-cultural adaptation of an existing instrument. Additional items were developed to address aspects related to the specific sociocultural conditions, regulatory context, and clinical practice of neonatal care in Greece. This process aimed at ensuring the content comprehensiveness and cultural appropriateness of the instrument under development. Initially, a pool of questions was generated by the questionnaire development team (R.S., M.L., and N.I.) to address the key thematic areas considered relevant to parental informed consent and decision-making regarding neonatal research participation. The candidate items were reviewed iteratively by the development team for relevance, clarity, overlap, and appropriateness for the Greek neonatal context. During this process, overlapping or redundant items were removed or merged, wording was refined where necessary, and additional items were introduced when specific thematic aspects were considered insufficiently represented.

Following a series of discussions within the development team, the most relevant and appropriate items were selected, and the preliminary version of the questionnaire was thus developed. The resulting set of items constituted the preliminary version of the questionnaire submitted to the expert panel for content evaluation.

The final instrument was organized into five domains:

(a) Demographic characteristics;
(b) Knowledge and understanding of clinical research;
(c) The informed consent process;
(d) Trust in healthcare professionals;
(e) Emotional responses related to participation in clinical research.

Responses were recorded using a combination of dichotomous variables, multiple-choice questions, and five-point Likert-scale items. Items were intended to be interpreted individually within their respective thematic domains rather than combined into a total questionnaire score. For Likert-type items, higher response values reflected greater agreement with the respective item; however, no overall or domain-level score was intended to represent a single underlying construct.

### 2.3. Ethical Approval and Informed Consent

The study was conducted in accordance with the ethical principles of the Declaration of Helsinki and its subsequent amendments ensuring participant confidentiality throughout the study, the right to informed consent and entirely voluntary participation, and finally the right to withdraw from the study at any time without any adverse consequences.

Data collection was conducted between September 2025 and December 2025 and included parents of newborns delivered at the Department of Obstetrics and Gynaecology and hospitalized in the Neonatology Department of the National and Kapodistrian University of Athens (NKUA) at Aretaieio University Hospital.

Participants received comprehensive verbal and written information regarding the purpose and nature of the study, the participation procedures, and the potential benefits and limitations of the research. Completion and return of the questionnaire following provision of this information constituted informed consent to participate in the study.

Participation was voluntary, and parents could withdraw from the study at any time before the final submission of the questionnaire.

Strict confidentiality was maintained throughout all stages of the study. Data processing and storage were conducted in accordance with the European Union General Data Protection Regulation (GDPR 2016/679) and the relevant Greek legislation on the protection of personal data. Study findings were reported only in aggregate form, without directly identifying information. Prior to study initiation, the research protocol received approval from the Scientific Board and the Research Ethics Committee of Aretaieio University Hospital (No: 704/24.07.2025).

Questionnaire data were pseudonymized using unique participant identification codes. No names or other directly identifying information were recorded on the questionnaires or entered into the analytical database. Initially, one member of the research team (R.S.) provided each parent with detailed verbal information regarding the purpose and methodology of the study. Parents who agreed to participate were then provided with a paper-based version of the questionnaire, which they completed independently without assistance from the research team. Each participant was assigned a unique identification code by R.S., with corresponding codes used for the first (Xa) and second (Xb) administrations, allowing responses from the two time points to be matched for the test–retest analysis without recording participants’ names or other directly identifying information on the questionnaires. The correspondence between the identification codes and participants was known only to R.S. and was not accessible to the other members of the research team. Questionnaire responses were subsequently entered into the study database by other research team members, who had access only to the coded questionnaires and were unaware of the participants’ identities.

### 2.4. Content Validity Assessment

The content validity of the preliminary questionnaire was evaluated using a modified Delphi technique. The Delphi method is appropriate for a systematic gathering of expert opinions and typically involves a series of structured stages [14,15].

In the present study, a modified two-stage approach was employed, consisting of an initial structured quantitative and qualitative expert assessment of the questionnaire followed by revision based on the experts’ feedback and subsequent expert review of the revised version by the same expert panel. Keeney et al. [14] define experts as “informed individuals,” “specialists in their field,” and people possessing relevant knowledge and expertise in the subject under investigation. Accordingly, an expert panel was assembled. The expert panel comprised five members with complementary clinical, academic, and methodological expertise relevant to neonatal research and questionnaire development. Two were professors of paediatrics–neonatology (Z.I. and T.B.), and two were paediatricians–neonatologists (M.T. and A.K.), each with more than 20 years of clinical experience in neonatology. The fifth member was a biostatistician (A.P.) with expertise in research methodology and questionnaire development. Experts were purposively selected on the basis of their relevant clinical, academic, methodological, and research expertise. All five invited experts participated in both stages of the process, corresponding to a response rate of 100%. During the first stage, the experts received the preliminary questionnaire together with a content evaluation form and information describing the purpose of the instrument and the evaluation procedure. Each expert independently evaluated every questionnaire item and was invited to provide qualitative comments regarding its clarity, relevance, representativeness, and appropriateness for the target population. Specifically, each item was evaluated with respect to clarity of wording according to the following criteria:

- Relevance to the construct under investigation;
- Representativeness of the content;
- Appropriateness for the target population.

Item evaluation was performed using a four-point Likert scale, with response options ranging from 1 (not relevant) to 4 (highly relevant).

Content validity was assessed using the Content Validity Index (CVI). For each questionnaire item, the Item-Level Content Validity Index (I-CVI) was calculated as the proportion of experts who rated the item as either 3 (quite relevant) or 4 (highly relevant) divided by the total number of experts. Consistent with established recommendations for content validation studies. For a five-member expert panel, an I-CVI of 1.00 was considered acceptable, corresponding to agreement among all five experts regarding item relevance [16]. All five experts rated all questionnaire items as either 3 or 4 during the initial quantitative assessment, resulting in an I-CVI of 1.00 for each item.

In addition, the Scale-Level Content Validity Index based on the average approach (S-CVI/Ave) was calculated to assess the overall content validity of the questionnaire. To account for chance agreement among experts, a modified kappa statistic was additionally calculated for each item based on the probability of chance agreement.

Despite complete quantitative agreement regarding item relevance, the experts provided qualitative comments and suggestions, primarily concerning linguistic and wording refinements aimed at improving item clarity. These recommendations were reviewed by the questionnaire development team and incorporated where appropriate. The modifications did not alter the conceptual content or intended thematic focus of the items.

Following incorporation of these modifications, the revised questionnaire was returned to the same expert panel for a second-stage review. During this stage, the experts reviewed the revised wording and confirmed that the modifications appropriately reflected the recommendations provided during the initial assessment. No additional quantitative rating was performed during the second stage, as the initial quantitative assessment had already demonstrated complete agreement and the subsequent modifications were limited to linguistic and wording refinements without changes to item content.

### 2.5. Pilot Testing

#### Pilot Testing in a Small Representative Sample of Healthcare Professionals and Assessment of Test–Retest Reliability

Following completion of the expert review process, the questionnaire was pilot tested in a sample of parents of newborns. The objectives of the pilot study were to:

- Assess the comprehensibility of the questions;
- Identify potential ambiguities in item wording;
- Evaluate participants’ acceptance of the instrument;
- Estimate the time required for questionnaire completion;
- Assess the functionality of the data collection procedure.

To assess the temporal stability of participants’ responses, the test–retest method was employed [17]. The procedure consisted of the following steps:

1. Initial administration (test): The questionnaire was administered to a representative sample of participants.
2. Retest interval: An appropriate time interval was allowed between the two administrations in order to minimize the likelihood of participants to recall their previous responses while ensuring that no substantial change in the underlying constructs being measured had occurred. This interval was set to be longer than two weeks and shorter than six weeks.
3. Second administration (retest): The same questionnaire was re-administered to the same participants.

Participants completed the questionnaire independently in written form and were provided with an open-ended field in which they could freely report comments or suggestions regarding the clarity, comprehensibility, wording, and overall completion of the questionnaire. No interviews or formal cognitive debriefing sessions were conducted. Following completion of the pilot testing phase, the written feedback was reviewed descriptively by the questionnaire development team to identify potential difficulties in comprehension, ambiguous wording, or suggestions for improvement. No formal qualitative coding or thematic analysis was performed. Following completion of the pilot testing phase, participant feedback was reviewed, and minor linguistic and formatting modifications were made where considered necessary. This procedure was intended to inform the preliminary refinement of the questionnaire and was not designed as a formal qualitative validation study. Given the multidomain descriptive nature of the questionnaire and the absence of a predefined total or domain-level score, test–retest reliability was primarily evaluated and interpreted at the individual-item level. As this was an exploratory pilot phase, the sample was intended to provide preliminary information on questionnaire comprehensibility, feasibility, and the temporal stability of individual items rather than to support definitive psychometric validation or assessment of the dimensional structure of the instrument.

### 2.6. Statistical Analysis

All statistical analyses were performed using the R statistical software environment (version 4.5.1) [18]. Test–retest reliability for ordinal and Likert-type items was assessed using the weighted kappa from irrCAC package [19] version 1.4 using linear weights.

For other unordered categorical items, test–retest reliability was evaluated using the standard Cohen’s kappa coefficient (κ); furthermore percentage agreement was also provided for the demographics items.

The results for Kappa values were interpreted as follows:

- Values < 0.40 indicated poor agreement;
- Values between 0.40 and 0.59 indicated moderate agreement;
- Values between 0.60 and 0.79 indicated good agreement;
- Values ≥ 0.80 indicated excellent agreement.

Percentage agreement was interpreted according to the following criteria:

- Values < 60% indicated poor agreement;
- Values between 60% and 74% indicated moderate agreement;
- Values between 75% and 84% indicated good agreement;
- Values ≥ 85% indicated very good agreement.

The completeness of questionnaire responses was assessed before analysis. No item-level missing data were identified among the 20 participants included in the test–retest analysis; therefore, no imputation procedures were required.

## 3. Results

### 3.1. Questionnaire Development

The questionnaire development process, including initial item development by the questionnaire development team followed by expert panel evaluation and subsequent refinement, resulted in a 33 item questionnaire. The complete questionnaire is provided in the Supplementary Materials, including both the original Greek version and an English translation, together with all items and corresponding response options. The instrument included a combination of a five-point Likert scale ranging from 1 (strongly disagree) to 5 (strongly agree), dichotomous (yes/no) questions, and multiple-choice questions (including those assessing demographic information).

### 3.2. Questionnaire Domains

The developed questionnaire consists of five distinct domains and a total of 33 items, which were designed with an aim to fully investigate parental consent for neonatal participation in research protocols and the factors influencing parental decision-making.

#### 3.2.1. Domain A: Demographic Characteristics

The first domain collects participants’ demographic information, including sex, age, ethnicity, religious affiliation, educational attainment, professional involvement in the healthcare sector, relationship to the newborn, and the presence of other children in the family.

#### 3.2.2. Domain B: Information and Understanding of Clinical Research

The second domain assesses participants’ knowledge and understanding of clinical research, including their ability to distinguish between clinical care and research activities, as well as their attitudes toward the necessity of clinical research. It also includes items exploring the influence of study type (interventional versus observational) on decision-making for consent and participants’ preferred ways of providing information.

#### 3.2.3. Domain C: Informed Consent Process

The third domain focuses on parents’ experiences during the informed consent process, including their understanding of the consent form, the time available to make a decision, their perceived freedom to decline participation, the opportunity to ask questions, and the emotional responses experienced during the decision-making process.

#### 3.2.4. Domain D: Trust in and Relationship with Healthcare Professionals

The fourth domain examines participants’ level of trust in the physicians and nursing staff of the NICU, the extent to which they perceive their views and preferences to be respected, their sense of security during the information and consent process, and their preferences regarding the source of information on research-related matters.

#### 3.2.5. Domain E: Emotions Regarding Participation in Clinical Research

The fifth domain explores parents’ emotional responses to the potential participation of their newborn in a research study including feelings of anxiety, fear, trust, hope, decision-making pressure, and the overall emotional burden associated with the informed consent process and research participation.

### 3.3. Content Validity Test

The quantitative content validity assessment conducted during the first stage of the expert review showed that all individual questionnaire items achieved an I-CVI of 1.00, indicating complete agreement among the expert panel regarding the relevance and appropriateness of each item included in the instrument. The corresponding modified kappa coefficient was also 1.00 for each item, indicating excellent agreement beyond chance. Furthermore, the S-CVI/Ave was also 1.00, demonstrating excellent overall content validity for the preliminary questionnaire.

In addition to the quantitative assessment based on the CVI, the expert panel provided qualitative feedback on the questionnaire. The main recommendations focused on improving the wording of several items to enhance clarity, as well as incorporating neutral response options (e.g., “neither agree nor disagree”) where deemed necessary. Furthermore, experts suggested restructuring the sequence of questionnaire items to improve the overall flow and respondent experience, particularly in the paper-based version of the instrument. Following incorporation of these recommendations, the revised questionnaire was returned to the same expert panel for second-stage review, during which the experts confirmed that the modifications appropriately reflected their initial recommendations.

### 3.4. Test–Retest Reliability

During the study period, 78 neonates were born at the study centre. Parents of 10 neonates were not considered eligible for participation because of insufficient proficiency in the Greek language to complete the questionnaire. Among the 68 eligible families, 15 declined participation, while 53 parents consented to participate and completed the questionnaire at the initial assessment. Of these, 20 returned for scheduled clinical follow-up within the predefined test–retest interval and completed the questionnaire for a second time, thus constituting the final sample for the test–retest analysis. The remaining 33 participants were not included in the test–retest assessment because they did not attend a follow-up visit within the predefined study interval. The test–retest reliability of the questionnaire was evaluated through repeated administration of the instrument to a sample of 20 parents whose newborns were delivered at the Department of Obstetrics and Gynaecology and subsequently hospitalized in the Neonatal Clinic of Aretaieio University Hospital.

The pilot sample consisted of 20 participants, including 10 women (50%) and 10 men (50%). Participants were recruited based on their availability and willingness to participate through convenience sampling.

Test–retest reliability was evaluated at the individual-item level, with results organized according to the thematic domains of the questionnaire. For categorical variables, percentage agreement between the two administrations and Cohen’s kappa coefficient (κ) were calculated. For ordinal/Likert-type variables, reliability was assessed using weighted kappa. The results are presented graphically for each questionnaire item, indicating the statistical measure applied (Cohen’s κ or weighted kappa and for the demographic data percentage of agreement), together with a color-coded classification of the level of agreement. The five domains were used to organize conceptually related items and were not intended to represent empirically established psychometric subscales. Items are interpreted individually within their respective thematic domains, and no domain-level or total score is calculated. For Likert-type items, response values are interpreted according to the wording and direction of each individual item rather than according to a uniform positive–negative scoring scheme.

#### 3.4.1. Domain A: Demographic Characteristics

All items in this domain were categorical variables. There was perfect agreement across all demographic items, with a percentage agreement of 100% (95% CI = 83.9–100%) and kappa = 1 (95% CI 1–1).

#### 3.4.2. Domain B: Information and Understanding of Clinical Research

**Figure 1:**
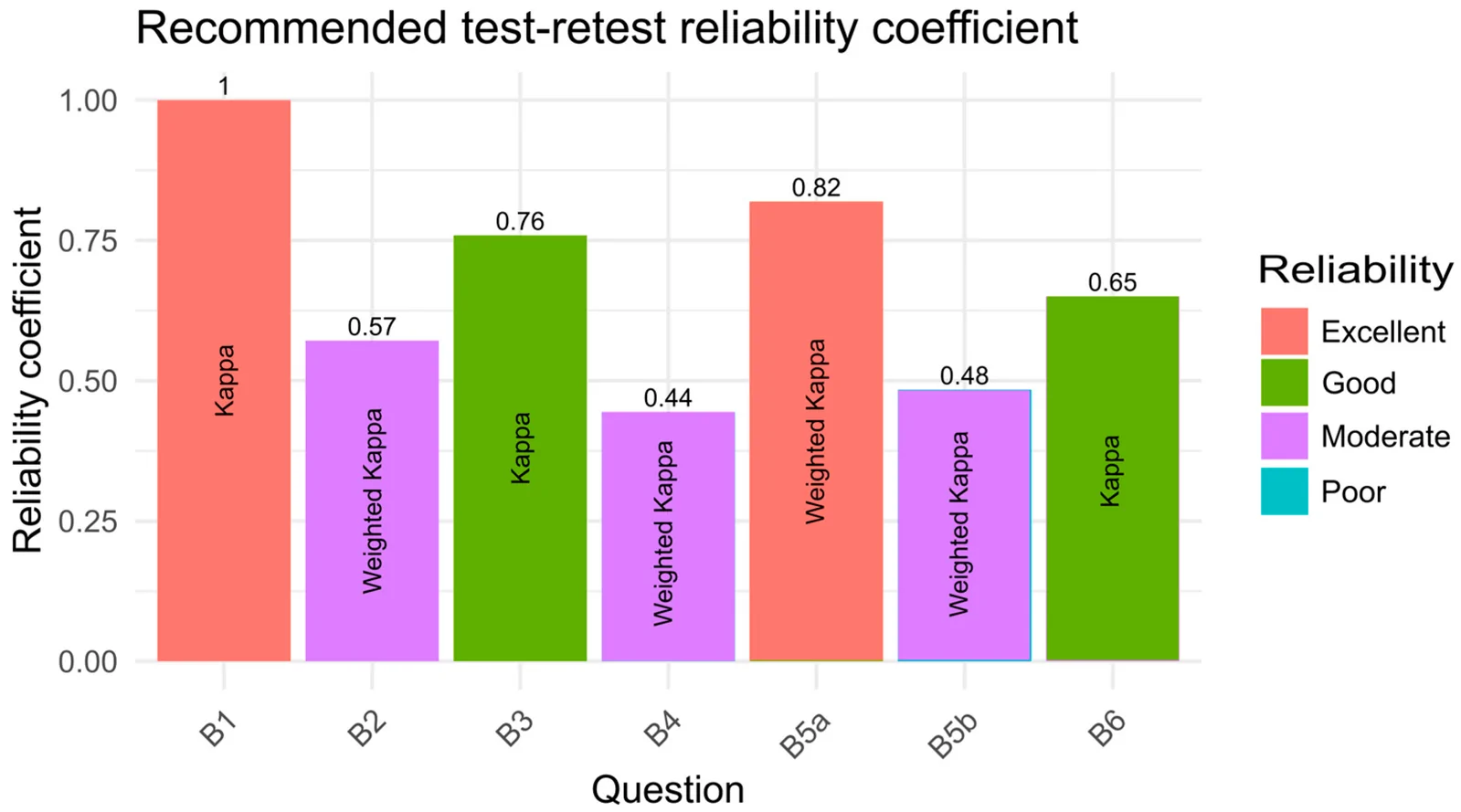
Test–Retest Reliability Results for Items Assessing Information and Understanding of Clinical Research.

Items included in Domain B demonstrated moderate-to-excellent test–retest reliability (Table 1, Figure 1). Question B1 (“Have you previously heard about clinical studies involving newborns?”) demonstrated excellent agreement, with kappa being 100%, which indicates complete stability of responses between the two assessment time points. Excellent agreement was also observed for B5a (kappa = 0.82), while questions B3 and B6 demonstrated good agreement. These items assessed participants’ understanding of the distinction between clinical care and research, their attitudes toward therapeutic research interventions, and their preferences regarding the mode of information delivery.

Although Questions B2, B4, and B5b yielded lower agreement values compared with the remaining items in this domain, their reliability indices remained within acceptable ranges.

Overall, the findings support satisfactory stability of the items included in Domain B and confirm their suitability for assessing parents’ level of information and understanding regarding clinical research in the neonatal setting (Table 1, Figure 1).

#### 3.4.3. Domain C: Informed Consent Process

Within Domain C, Questions C2–C6 were conditional follow-up items addressed only to participants who responded positively to Question C1 regarding previous experience of being asked to provide consent for their child’s participation in a research study. As two participants reported having such prior experience in the first round and one additional in the second round, Questions C2–C6 were mostly not completed and, therefore, could not be included in a test–retest reliability analysis.

The only item in this domain that could be evaluated was Question C1 (“Have you ever been asked to provide consent for your child’s participation in a research study?”). This item demonstrated good test–retest reliability, with a Cohen’s kappa coefficient of 0.772 (95% CI: 0–1) and a percentage agreement of 95% (95% CI: 76.4–99.1%); one participant reported initially no and during the second round yes.

#### 3.4.4. Domain D: Trust and Relationship with Healthcare Professionals

**Figure 2:**
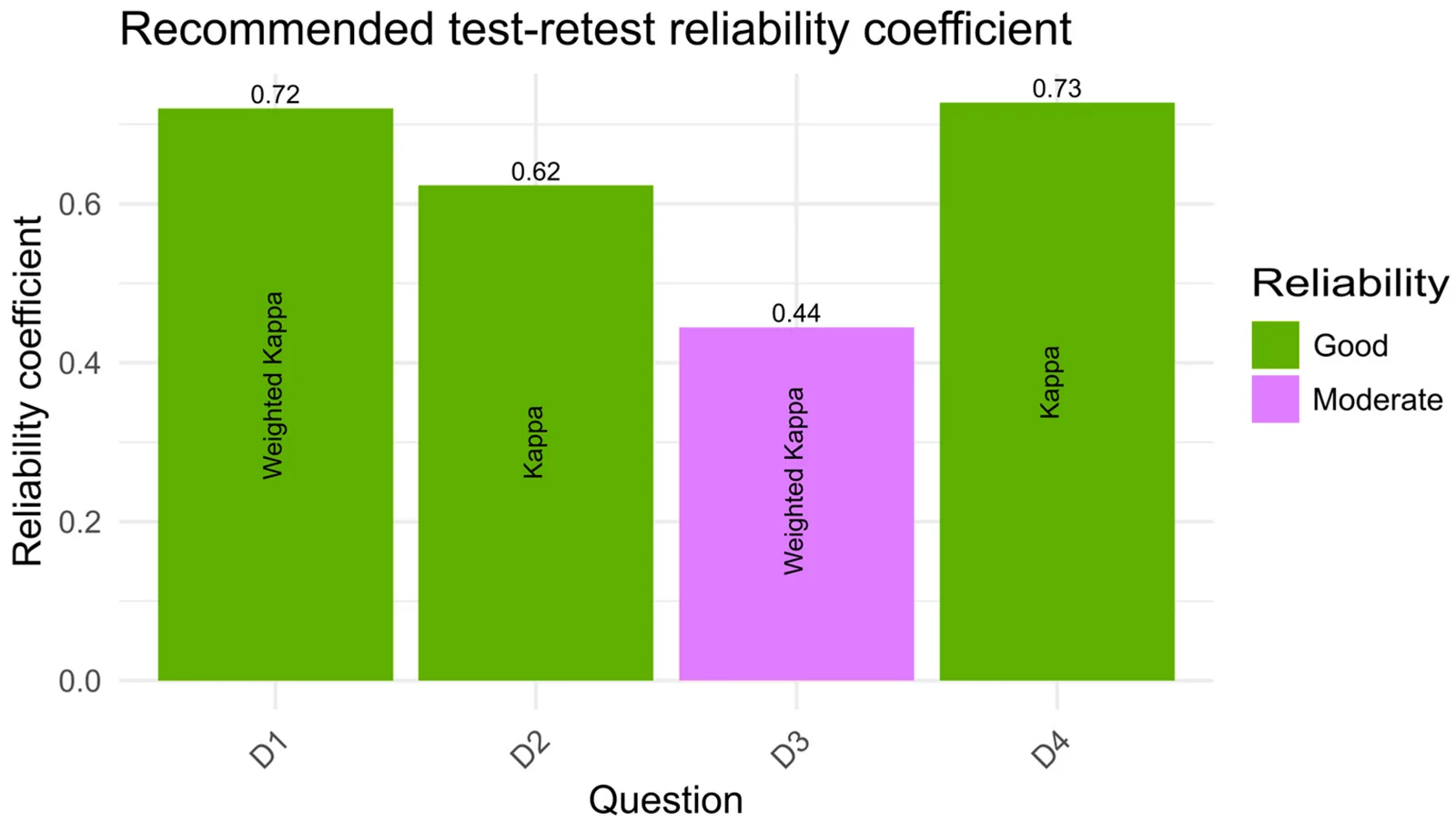
Test–Retest Reliability Results for Items Assessing Parents Trust and Relationship with Healthcare Professionals.

Overall, the items included in Domain D demonstrated moderate-to-good test–retest agreement between the two assessment time points (Table 2, Figure 2).

Questions D1 (“How much do you trust the medical staff of the NICU?”), D2 (“Do you feel that healthcare professionals respected your views and opinions?”), and D4 (“From whom would you prefer to receive information about research-related issues?”) demonstrated good test–retest agreement, with kappa ranging from 0.63% to 0.73%.

In contrast, Question D3 (“How well informed did you feel about your child’s condition and about whether and how the study was related to your child’s health?”) demonstrated lower stability across the two assessment time points. More specifically, the weighted kappa coefficient was 0.44, indicating moderate agreement.

#### 3.4.5. Section E: Emotions Related to Participation in a Research Study

**Figure 3:**
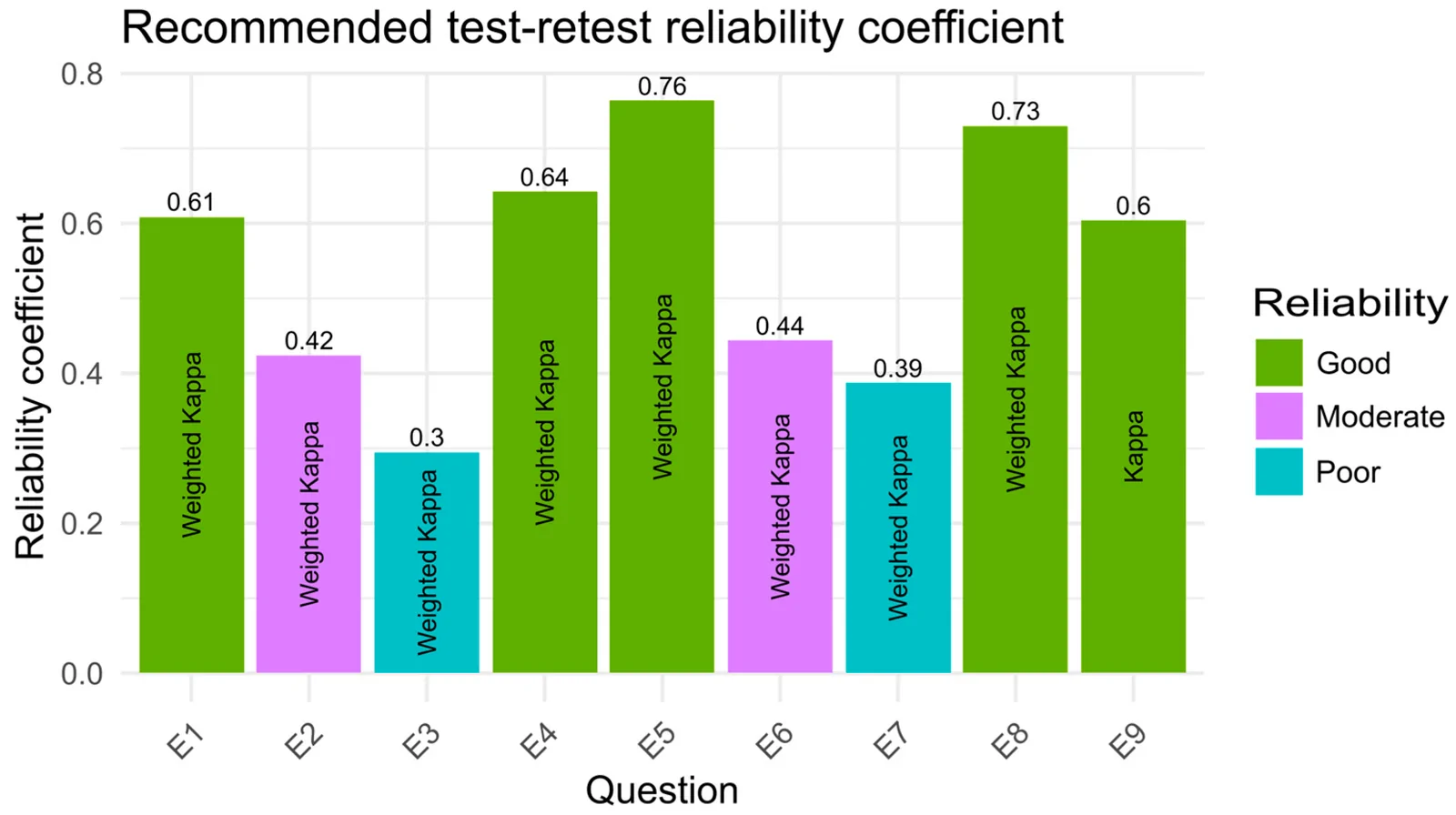
Test–Retest Reliability Results for Items Assessing Parental Emotional Responses.

The items in Section E demonstrated test–retest agreement ranging from poor to good across the two measurement time points (Table 3, Figure 3).

Items E5 and E8 showed the highest weighted kappa coefficients within this section. Specifically, item E5 (“I would feel an excessive emotional burden in making the decision to participate”) demonstrated good agreement (kappa = 0.76) as did item E8 (“I would feel annoyed if I were asked at this moment for my child to participate in a research study”) (kappa = 0.73).

Items E2, E3, E6 and E7 (“I am afraid to give consent, but I would feel guilty refusing and potentially depriving my child of possible benefits from the study”), which addressed hypothetical emotional responses, such as decision-related anxiety, enthusiasm about potential study participation, and feelings of pressure to make an immediate decision, demonstrated poor agreement. The lower agreement observed for some items indicates greater temporal variability in responses to specific hypothetical emotional situations.

### 3.5. Participant Feedback During Cognitive Debriefing

As part of the pilot testing process, participants were invited to provide structured feedback regarding the clarity, comprehensibility, wording, and practical completion of the questionnaire. This cognitive debriefing was intended to identify potential difficulties in understanding or completing individual items and was not designed or analysed as a formal qualitative interview study. Participants generally reported that the questionnaire was understandable and feasible to complete. The average completion time ranged between 10 and 15 min. None of the participants reported significant difficulties in understanding the questions or the available response options.

Participants’ feedback led to minor revisions in the wording of several items to improve clarity and reduce the potential for misinterpretation.

## 4. Discussion

The present paper reports the preliminary development and evaluation of a questionnaire designed to assess the attitudes, knowledge, and emotional responses of parents of neonates regarding their children’s participation in clinical research and the informed consent process. The need for the development of this instrument was motivated by the limited availability of validated and culturally adapted tools in the Greek context, as well as by the internationally recognized importance of informed consent in neonatal research.

The questionnaire was structured into distinct thematic domains informed by evidence from the international literature indicating that parents’ decisions regarding consent for their neonate’s participation in research are multifaceted and influenced by a combination of demographic, cognitive, emotional, and interpersonal factors.

Domain A, which included questions on parents’ demographic characteristics, was incorporated to explore factors that have been internationally associated with attitudes toward clinical research. Previous studies have demonstrated that parental age, educational level, cultural and ethnic background, migration status, religious beliefs, and prior parenting experience may influence both the understanding of research-related information and the willingness to provide informed consent [9,20,21,22,23,24]. Parents with higher levels of education tend to demonstrate a better understanding of research procedures and concepts, whereas cultural background and religious values frequently affect perceptions of potential risks and benefits associated with research participation [24,25].

When research involves neonates, the informed consent process must ensure that legally authorized representatives, typically the parents, receive sufficient and comprehensible information to make decisions regarding their child’s participation based on the child’s best interests [12]. In recent years, several international initiatives and regulatory frameworks, including the European Paediatric Regulation, the European Network for Paediatric Research at the European Medicines Agency (Enpr-EMA), and other international neonatal research networks, have sought to promote the conduct of safe and scientifically rigorous clinical research in neonatal populations [10,11].

The conduct of neonatal research varies considerably across countries, as the practice is affected by legal, ethical, and sociocultural contexts. The informed consent process provides a good example of this variability, as in the Netherlands, the participation of a neonate in a research protocol generally requires the consent of both parents, whereas in the United Kingdom, the consent of a single parent is typically considered sufficient. Similarly, in the United States, consent from both parents is usually required, although exceptions may apply in studies involving minimal risk or the prospect of direct clinical benefit to the child. In Belgium, conflicting interpretations of the relevant legislation regarding whether consent from one or both parents is required have created practical challenges for the recruitment of neonates into clinical studies [26,27]. For this reason, the distinction between mothers and fathers was considered relevant in the present questionnaire, as previous research has shown that mothers and fathers may differ in their perceptions of risks and benefits associated with research participation, especially when their infant is hospitalized in an NICU [28,29].

Domain B, which focused on information and understanding of clinical research, was designed to assess parents’ level of knowledge and understanding regarding clinical studies, their ability to distinguish between clinical care and research, their attitudes toward the necessity of clinical research, and the extent to which specific study characteristics influence their willingness to provide consent. The selection of these thematic areas was informed by an extensive body of international literature highlighting the understanding of the research process as one of the most important determinants of parental decision-making regarding children’s participation in clinical studies [20]. Previous studies have shown that adequate understanding of the research process, clear communication, and the ability to comprehend research-related information may facilitate informed decision-making, whereas limited comprehension and the complexity of the consent process may represent important barriers [30,31,32,33,34]. In the present study the item assessing self-perceived knowledge of clinical research demonstrated excellent-to-poor test–retest reliability, a finding that may reflect the subjective nature of self-assessment of knowledge. On the other hand, the item evaluating participants’ ability to distinguish between treatment and research showed high stability over time (kappa = 0.76), suggesting that this represents a more established cognitive construct among respondents. This distinction is particularly relevant, as a clear understanding of the distinction between clinical care and research is widely recognized in the literature as a fundamental prerequisite for valid informed consent [20].

In addition, the assessment of attitudes toward the necessity of clinical research was based on findings indicating that a positive perception of the contribution of research to medical progress and altruism are among the most common factors facilitating parental consent [20]. This item demonstrated moderate test–retest reliability (kappa = 0.44). The observed variability may reflect the attitudinal nature of the item, as perceptions regarding the role and value of clinical research may be less stable than factual knowledge. Furthermore, the international literature consistently highlights the phenomenon of “therapeutic misconception,” whereby parents conflate therapeutic care with research procedures and assume that participation in a study necessarily entails direct therapeutic benefit for their child. Previous research has consistently shown that parents’ willingness to enrol their child in clinical research is primarily shaped by their perception of the balance between potential risks and anticipated benefits, as well as by study-related characteristics, including study duration, participant burden, and methodological features such as randomization and placebo allocation [20,30,32,33,34,35,36,37].

Overall, the expectation of direct benefit to the child represents the strongest facilitator of participation, whereas concerns regarding potential risks, adverse effects, and the burden associated with study participation constitute the principal barriers to consent [30,32,33,34,35,36,37,38]. To capture these differences, the questionnaire included two separate items addressing parental willingness to consent to participation in interventional and observational studies. The item assessing consent for participation in an interventional study demonstrated excellent test–retest reliability (kappa = 0.82), while the item evaluating consent for participation in an observational study exhibited only moderate agreement (kappa = 0.48). Although this difference should be interpreted cautiously given the small pilot sample, it suggests that responses concerning willingness to participate may vary according to the type of research being considered.

Finally, an item addressing parents’ preferred modes of information delivery was included in view of increasing interest in alternative approaches to the informed consent process. Key recommendations for improving the informed consent process include antenatal information and discussion, appropriate training of individuals obtaining consent, continuous bidirectional communication, and the provision of clear, comprehensible, and appropriately tailored information [4,39,40]. Technological developments, including electronic informed consent approaches, may provide additional opportunities to support information delivery and parental engagement in research decision-making [13,41]. These approaches appear to improve comprehension of the information provided and to strengthen parental engagement in decision-making, particularly in settings where available time is limited.

Overall, the findings from the pilot sample provide preliminary evidence regarding the temporal stability of individual items within Domain B, with variability observed across items. Together with their grounding in the existing literature, these findings support the inclusion of these thematic areas in the questionnaire, while further evaluation in larger samples is required.

Domain C (Informed Consent Process) was designed to assess parents’ experiences during the informed consent process, focusing on comprehension of the consent form, sufficiency of decision-making time, opportunity to ask questions, perceived freedom of choice, and the emotions associated with the process. An initial item addressed participation of another child in the family in a clinical study, as previous research experience may influence parental trust and attitudes toward future research participation [42,43].

The inclusion of these domains was informed by the international literature, which highlights the quality of information and communication with the research team as critical determinants of valid parental consent [20]. Adequate time for decision-making has been shown to facilitate consent, whereas time constraints constitute a significant barrier [9,30,31]. Moreover, the clarity and presentation of information play a pivotal role, as parents with better understanding of the consent form, engagement in discussion, and a positive initial impression are more likely to provide consent [35].

In parallel, the assessment of parental emotions was considered essential, as fear, distress, and emotional burden have been identified as common barriers to children’s participation in clinical research. Obtaining informed consent from parents of critically ill neonates may be particularly challenging [2,7]. Parents may experience significant emotional strain, while mothers may face medical complications such as preeclampsia or recovery from anaesthesia [2,44]. Under these conditions, parents often feel overwhelmed and may have difficulty adequately understanding study-related information, especially in time-pressured settings [7,39]. Consequently, they may feel unable to make informed decisions regarding their infant’s participation in research [39].

It is worth noting that items C2–C6 were designed as conditional questions and were addressed exclusively to parents with prior experience of being asked to provide consent for their child’s participation in a research study.

At the initial assessment, only two participants reported having previously been asked to provide consent for their child’s participation in a research study, while one additional participant reported such experience at retest. Consequently, the conditional items C2–C6, which were applicable only to parents with such prior experience, were completed by an insufficient number of participants to allow a reliable assessment of their test–retest reliability. The measurement performance of these conditional items therefore remains undetermined in the present study. Nevertheless, these items were retained because they address important aspects of the informed consent experience identified in the international literature as potentially relevant to parental decision-making regarding neonatal research participation. Their measurement properties should be evaluated in future studies specifically including a larger number of parents with direct experience of the research consent process.

Domain D (Trust and Relationship with Healthcare Professionals) was designed to assess parents’ trust in healthcare professionals and the potential role of the parent–healthcare professional relationship in decision-making regarding participation in clinical studies. The selection of these items was based on findings from the international literature, according to which trust in the researcher or in the individual providing information constitutes one of the most important factors facilitating parental consent [9,32,35,45]. Similarly, trust in the medical team has been reported as a factor that enhances positive attitudes toward participation in research protocols [33]. Parents are more likely to provide consent when they feel that healthcare professionals respect their views, provide sufficient and comprehensible information, and act in the best interests of their child [33,34,35,36]. Conversely, lack of trust in researchers and negative interactions during the information process have been reported as significant barriers to obtaining consent [33,36,37,45]. At the same time, the perception of freedom in decision-making has been recognized as a fundamental prerequisite of valid informed consent [34], whereas feelings of pressure or obligation have been described both as factors leading to consent and as barriers to the decision-making process [33,34].

In the present pilot sample, items within Domain D showed varying levels of agreement between the two administrations. Items related to trust in NICU staff, respect for parental views, and preferences regarding the person providing information showed good levels of agreement (kappa = 0.62–0.73). These findings provide preliminary evidence of temporal stability for these individual items.

In contrast, the item assessing the adequacy of information provided regarding the child’s condition and its relation to the research study demonstrated lower stability (kappa = 0.44). The international literature indicates that parents’ perceptions of the quality and adequacy of information are not stable constructs but are influenced by their emotional state, their experiences during hospitalization, and their interactions with the healthcare team [35,36]. Therefore, the greater variability observed in this item may reflect the context-dependent nature of parents’ perceptions of information, which can be influenced by their experiences during hospitalization and interactions with the healthcare team rather than a limitation of the measurement instrument. However, the wording of item D3 should also be considered, as the item incorporates more than one informational component, which may have contributed to the lower temporal stability observed. Importantly, direct prior experience with research consent was limited in the present pilot sample, with only two participants reporting such experience at the initial assessment and one additional participant changing their response from “No” at the initial assessment to “Yes” at retest. Responses to D2 and D3 items may therefore have reflected a hypothetical interpretation or participants’ experiences of routine clinical communication rather than an actual research consent episode. This limited direct exposure may have contributed to the observed variability; however, given the small pilot sample, these findings should be interpreted cautiously, and the wording of these items warrants further evaluation and potential refinement in subsequent studies. Overall, the findings from Domain D highlight the relevance of including trust and communication with healthcare professionals when exploring parental perspectives on neonatal research participation. The variability observed across individual items further supports the need for their evaluation in larger and more diverse samples.

Domain E (Emotional Responses to Participation in a Research Study) was developed based on the premise that decision-making in the neonatal care setting is not solely a cognitive process. International studies have shown that anxiety, fear, uncertainty, guilt, hope, and time pressure significantly influence parental decision-making [46]. In particular, parents of neonates admitted to intensive care units often report conflicting emotions: on the one hand, they wish to protect their child from potential risks, while on the other, they fear depriving them of possible therapeutic opportunities [20].

In the present pilot sample, items addressing emotional responses showed greater variability between the two administrations than several items addressing cognitive aspects or research-related attitudes. This variability may reflect the context-dependent nature of emotional responses, which can fluctuate over time and may be influenced by individual circumstances and experiences at the time of assessment.

Despite this variability, certain emotions—such as the feeling of excessive emotional burden (E5, kappa = 0.76) and irritation in response to the request for participation (E8, kappa = 0.73)—demonstrated relatively high temporal stability. This suggests that more immediate and unidimensional emotional reactions tend to remain more consistent over time. However, the interpretation of item E8 should also take into account its explicitly time-dependent wording (“at this moment”), which may influence responses across different assessment time points and warrants further evaluation. In contrast, item E7, which combined fear and guilt, exhibited lower test–retest reliability (kappa = 0.39), likely reflecting the cognitive and emotional conflict arising from the simultaneous evaluation of potential risks and possible benefits for the child. At the same time, the inclusion of two distinct emotional responses-fear and guilt-within a single item may have contributed to response variability, suggesting that the wording and structure of this item warrant further evaluation and potential refinement.

In the international literature, fear and emotional burden are consistently identified as key barriers to parental consent, with frequent references to concerns that the child may be treated as a “guinea pig” [33,36,37,45]. In the present study, the variability observed in several emotionally focused items further indicates the need to examine these items in larger and more diverse samples. The relative instability of responses to items in this section may be attributed to the fact that they capture hypothetical rather than actual experiences, which are more susceptible to change depending on the respondent’s cognitive framing and emotional state at the time of completion. However, this remains a hypothesis, and the present pilot design cannot distinguish true changes in emotional state from variability attributable to the measurement properties of the items. Consequently, lower test–retest coefficients should not be interpreted as evidence of adequate measurement performance simply because the constructs assessed are emotionally or contextually sensitive. Future studies involving parents with direct experience of the informed consent process together with further cognitive evaluation of item wording, will be important for evaluating these items under real-world conditions. Overall, the findings suggest that individual items addressing emotional and hypothetical situations may exhibit greater variability compared to those assessing more stable attitudes or perceptions. These findings provide preliminary evidence that emotional responses related to informed consent may be dynamic and influenced by multiple contextual and individual factors. At the same time, the variability observed across individual items identifies specific aspects that may require further evaluation and refinement. Nevertheless, the inclusion of emotional responses remains conceptually important when assessing parental perspectives on neonatal research participation, as a better understanding of these responses may contribute to the development of more supportive and family-centred informed consent processes. Further evaluation in larger and more diverse populations is warranted.

The findings of the present study are consistent with the core theoretical assumption underlying the questionnaire, namely that parental decision-making regarding the participation of a neonate in a clinical study is a multidimensional and dynamic process. This decision is not determined solely by knowledge but is also influenced by trust, emotional state, and the perceived balance between risks and benefits.

The greater variability observed in some items addressing attitudes and emotional responses may reflect the context-dependent nature of these dimensions, particularly in neonatal care settings, where parents’ experiences perceptions and emotional responses can change over time. These findings highlight the importance of considering the potential temporal variability of such responses when evaluating parental perspectives on research participation. The variability observed across individual items also underscores the importance of considering the nature and response format of each item when evaluating temporal stability. In a multidomain questionnaire addressing cognitive, attitudinal, relational, and emotional aspects, different items may exhibit different patterns of stability over time. The use of a test–retest design in a population of parents of neonates is methodologically demanding, as hospitalization conditions the circumstances of hospitalization, and parents’ experiences over time may influence their responses, thereby partially limiting observed stability.

The study has several limitations. The sample size in the pilot phase was small, which limits the precision and generalizability of the test–retest estimates and precludes definitive conclusions regarding the measurement properties of the questionnaire. In addition, the use of convenience sampling may have introduced selection bias. Therefore, the findings should be interpreted as preliminary and require confirmation in larger and more diverse populations.

Second, the study was conducted at a single hospital centre, which may limit the applicability of the findings to parents from other clinical and sociocultural settings. The third limitation has to do with the fact that some items assess hypothetical scenarios, which do not necessarily reflect actual behaviour in real-life decision-making contexts. Furthermore, the evaluation was conducted in a single hospital centre, which may limit the generalizability of the findings. In addition, only a very small number of participants had prior experience of being approached for consent to their child’s participation in research, precluding meaningful test–retest evaluation of the conditional items (C2–C6) in Domain C. Consequently, the measurement performance of these items remains undetermined and requires evaluation in future studies including a larger number of parents with direct experience of the research consent process. Finally, given the preliminary nature of the study and the small sample size, a more comprehensive evaluation of the questionnaire’s measurement properties was beyond the scope of the present study. In addition, the pilot evaluation identified specific items with potentially complex, multidimensional, or time-dependent wording, which may have influenced their temporal stability. These items require further cognitive evaluation and potential refinement, followed by reassessment in a larger sample. Furthermore, the 2–6-week test–retest interval may represent a limitation for the assessment of state-like emotional constructs, as genuine changes in parental emotional state or in the infant’s clinical condition may occur during this period, making it difficult to distinguish true temporal changes from measurement-related variability. Further internal validation in a larger and more diverse sample is therefore required, followed by external validation in independent populations before broader application of the questionnaire.

## 5. Conclusions

The present study describes the preliminary development and evaluation of a questionnaire designed to assess parental attitudes, knowledge, perceptions, and emotional responses regarding neonatal participation in clinical research and the informed consent process. The development of the instrument was motivated by the limited availability of tools specifically addressing these aspects within the Greek neonatal research context, as well as by the internationally recognized importance of informed consent in neonatal research.

The variations observed across the different thematic domains highlights the complex and multidimensional nature of parental decision-making regarding neonatal research participation. In the present pilot sample, items addressing cognitive aspects generally showed greater temporal stability, whereas items related to emotional responses exhibited greater variability between assessments. These findings should be interpreted cautiously given the preliminary nature of the study and the limited sample size. The conceptual structure of the instrument is informed by the international literature, which recognizes that informed consent is not an exclusively cognitive process but rather emerges from the interaction of knowledge, attitudes, emotions, and the relationship of trust with healthcare professionals. By incorporating these different thematic areas, the questionnaire provides a structured framework for exploring factors potentially associated with parental decision-making regarding neonatal research participation within the Greek context.

Overall, the present study represents an initial step in the development and evaluation of a questionnaire addressing parental perspectives on neonatal research participation and informed consent in Greece. The preliminary findings provide evidence regarding the content validity and temporal stability of individual questionnaire items while also identifying items and thematic areas that may require further evaluation or refinement. Further assessment in larger and more diverse populations is required to comprehensively establish the measurement properties of the questionnaire. The next phase of its development will involve comprehensive internal validation in an adequately powered Greek population, allowing further refinement of the instrument based on the resulting psychometric evidence. This should subsequently be followed by external validation in independent Greek populations and, ultimately, by cross-cultural evaluation and validation in international populations to determine its generalizability and applicability across different clinical, cultural, and healthcare settings.

## Figures and Tables

**Table 1:** Test–Retest Reliability of Items in Domain B: Information and Understanding of Clinical Research.

Item	Title	Agreement	95% CI of Agreement	Level of Repeatability
B1	Have you previously heard about clinical studies involving newborns?	1	1–1	Excellent
B2	How would you describe your level of knowledge about clinical research studies?	0.57	0.28–0.87	Moderate
B3	In your opinion, what is the main difference between medical treatment and a clinical research study?	0.76	0.21–1	Good
B4	Do you believe that clinical research studies are necessary for improving medical treatments?	0.44	0.12–0.77	Moderate
B5a	Do you believe that the type of research study influences your decision to provide consent for your child’s participation? (a) If the study involved a therapeutic intervention (e.g., administration of a medication or implementation of a new treatment approach), how likely would you be to provide your consent for your child’s participation?	0.82	0.62–1	Excellent
B5b	(b) If the study were observational and involved only the collection of data (with no intervention affecting your child), how likely would you be to provide your consent for your child’s participation?	0.48	0.18–0.79	Moderate
B6	Which form of information would be most helpful to you?	0.65	0.38–0.92	Good

**Table 2:** Test–Retest Reliability Results for Domain D: Trust and Relationship with Healthcare Professionals.

Item	Title	Agreement	95% CI of Agreement	Level of Repeatability
D1	How much do you trust the medical staff of the NICU?	0.72	0.43–1	Good
D2	Do you feel that healthcare professionals respected your views and opinions?	0.62	0.14–1	Good
D3	How well informed did you feel about your child’s condition and about whether and how the study was related to your child’s health?	0.44	0.11–0.78	Moderate
D4	From whom would you prefer to receive information about research-related issues?	0.73	0.44–1	Good

Abbreviation: neonatal intensive care unit (NICU).

**Table 3:** Repeatability Test Results for Emotion-Related Questions.

Item	Title	Agreement	95% CI of Agreement	Level of Repeatability
E1	I would feel anxious if I were approached to participate in this research study	0.61	0.37–0.85	Good
E2	I would feel anxious about deciding whether my child should participate in the research study	0.42	0.18–0.67	Moderate
E3	I would feel enthusiastic about the opportunity for my child to participate in the research study	0.3	−0.03–0.62	Poor
E4	I would be afraid of the potential risks associated with my child’s participation in the research study	0.64	0.37–0.92	Good
E5	I would feel an excessive emotional burden in making the decision to participate	0.76	0.55–0.98	Good
E6	I would feel pressure to make an immediate decision regarding my child’s participation	0.44	0.07–0.82	Moderate
E7	I am afraid to give consent, but I would feel guilty refusing and potentially depriving my child of possible benefits from the study	0.39	0.07–0.71	Poor
E8	I would feel annoyed if I were asked at this moment for my child to participate in a research study	0.73	0.5–0.96	Good
E9	Would you feel comfortable declining without the risk of your child’s care being affected?	0.6	0.3–0.91	Good

## Data Availability

The data supporting the conclusions of this article will be made available by the authors on request.

## Supplementary Materials

The following supporting information can be downloaded at https://www.mdpi.com/article/10.3390/children13091227/s1: the complete questionnaire, including both the original Greek version and an English translation, together with all items and corresponding response options.
