# Supplementary material for: Development and Preliminary Evaluation of a Questionnaire on Parental Informed Consent in Neonatal Research
Source: Children (Basel). 2026 Sep 10;13(9):1227. doi: 10.3390/children13091227 (PMC13605765; doi:10.3390/children13091227)
Supplement: Supplementary file 1 [file children-13-01227-s001.zip › children-4475732-supplementary.pdf]

ΕΡΩΤΗΜΑΤΟΛΟΓΙΟ

Ενότητα Α: Δημογραφικά Στοιχεία

1. **Φύλο:** ☐ Άνδρας ☐ Γυναίκα
2. **Ηλικία:** ☐ <25 ☐ 25–34 ☐ 35–44 ☐ ≥45
3. **Καταγωγή:**
   Ελληνική ☐ Άλλη ☐ Αναφέρετε ποια…………..
4. **Θρήσκευμα:**
   Χριστιανός {ορθ./καθολ./προτεστ.) ☐ Μουσουλμάνος ☐ Άθεος ☐ Άλλο ☐
5. **Εκπαιδευτικό επίπεδο:**
   ☐ Υποχρεωτική ☐ Δευτεροβάθμια ☐ Ανώτερη/Ανώτατη ☐ Μεταπτυχιακό+
6. **Επάγγελμα σχετιζόμενο με τον τομέα υγείας/ έρευνα**
   Ναι ☐ Όχι ☐ .
   Αν η απάντηση είναι "Ναί" παρακαλώ διευκρινίστε:
   Π.χ. νοσηλευτής/τρια, ιατρός, φαρμακοποιός, κ.λπ.)
   ……………………………………..

7. **Σχέση με το νεογνό:** ☐ Μητέρα ☐ Πατέρας ☐ Άλλο
8. **Έχετε άλλα παιδιά;** ☐ Ναι ☐ Όχι

Ενότητα Β: Πληροφόρηση – Κατανόηση Κλινικής Έρευνας

1. **Έχετε ακούσει στο παρελθόν για κλινικές μελέτες σε νεογνά;**
   ☐ Ναι ☐ Όχι
2. **Πώς θα περιγράφατε τη γνώση σας για τις κλινικές μελέτες;**
   ☐ Καθόλου ☐ Λίγη ☐ Μέτρια ☐ Καλή ☐ Πολύ καλή
3. **Κατά τη γνώμη σας, ποια είναι η βασική διαφορά μεταξύ θεραπείας και μελέτης;**
   ☐ Η μελέτη είναι ερευνητική – δεν παρέχει άμεσα οφέλη
   ☐ Η μελέτη είναι πειραματική θεραπεία
   ☐ Δεν γνωρίζω
4. **Πιστεύετε ότι οι κλινικές μελέτες είναι απαραίτητες για τη βελτίωση των θεραπειών;**
   Διαφωνώ απόλυτα ☐ Διαφωνώ ☐ Ούτε συμφωνώ ούτε διαφωνώ ☐ Συμφωνώ ☐ Συμφωνώ απόλυτα ☐
5. **Πιστεύετε ότι το είδος της ερευνητικής μελέτης επηρεάζει την απόφασή σας να δώσετε συγκατάθεση για συμμετοχή του παιδιού σας;**

α) Αν η μελέτη αφορούσε μια θεραπευτική παρέμβαση (π.χ. χορήγηση φαρμάκου ή εφαρμογή νέας μεθόδου):

Πόσο πιθανό θα ήταν να δώσετε τη συγκατάθεσή σας;

☐ Καθόλου πιθανό ☐ Λίγο πιθανό ☐ Αρκετά πιθανό ☐ Πολύ πιθανό

β) Αν η μελέτη ήταν παρατηρητική και αφορούσε μόνο την καταγραφή στοιχείων (χωρίς καμία παρέμβαση στο παιδί σας):

Πόσο πιθανό θα ήταν να δώσετε τη συγκατάθεσή σας;

☐ Καθόλου πιθανό ☐ Λίγο πιθανό ☐ Αρκετά πιθανό ☐ Πολύ πιθανό

6. **Ποια μορφή ενημέρωσης θα σας βοηθούσε περισσότερο;**
☐ Έντυπο ☐ Προφορική ☐ Βίντεο ☐ Διάγραμμα ☐ Δεν έχει σημασία

Ενότητα Γ: Συναίνεση – Διαδικασία

1. **Σας έχει ζητηθεί συγκατάθεση για συμμετοχή σε μελέτη του παιδιού σας;**
☐ Ναι ☐ Όχι

   Εάν απαντήσατε «Ναι», παρακαλούμε προχωρήστε στις ακόλουθες ερωτήσεις. Εάν απαντήσατε «Όχι», παρακαλούμε προχωρήστε απευθείας στην Ενότητα Δ.

2. **Πόσο καλά κατανοήσατε το έντυπο συναίνεσης;**
☐ Καθόλου ☐ Λίγο ☐ Μέτρια ☐ Πολύ ☐ Απόλυτα
3. Ο χρόνος που σας δόθηκε για να αποφασίσετε ήταν:
☐ Ανεπαρκής ☐ Επαρκής ☐ Πλήρως ικανοποιητικός
4. **Νιώσατε άνετα να αρνηθείτε χωρίς τον κίνδυνο να επηρεαστεί η φροντίδα του παιδιού σας;**
☐ Ναι ☐ Όχι ☐ Δεν είμαι σίγουρος/η
5. **Σας δόθηκε δυνατότητα να ρωτήσετε;**
☐ Όχι ☐ Λίγο ☐ Ναι, πλήρως
6. **Ποια συναισθήματα κυριάρχησαν όταν σας ζητήθηκε συγκατάθεση; (επιλέξτε έως 2):**
☐ Άγχος ☐ Φόβος ☐ Εμπιστοσύνη ☐ Ελπίδα ☐ Δυσπιστία ☐ Ουδετερότητα

Ενότητα Δ: Εμπιστοσύνη – Σχέση με Επαγγελματίες Υγείας

1. **Πόσο εμπιστεύεστε το ιατρικό προσωπικό της ΜΕΝΝ;**
☐ Καθόλου ☐ Λίγο ☐ Μέτρια ☐ Πολύ ☐ Απόλυτα
2. **Αισθάνεστε ότι οι επαγγελματίες υγείας σέβονται τις απόψεις σας ως γονείς;**
☐ Ναι ☐ Όχι ☐ Εν μέρει
3. **Πόσο ασφαλείς νιώθετε με τον τρόπο που σας ενημερώνουν για την κατάσταση του παιδιού σας και για τη συσχέτιση τυχόν προγραμματισμένων ερευνητικών μελετών με την υγεία του;**
☐ Καθόλου ☐ Λίγο ☐ Μέτρια ☐ Πλήρως
4. **Από ποιον θα επιθυμούσατε να ενημερώνεστε για ερευνητικά θέματα;**
☐ Ιατρό ☐ Νοσηλευτή ☐ Ψυχολόγο ☐ Όλους ☐ Δεν με απασχολεί

Ενότητα Ε: Συναισθήματα σχετικά με την προσέγγιση για συμμετοχή σε ερευνητική μελέτη.

1. **Θα ένιωθα άγχος αν με πλησίαζαν για συμμετοχή σε αυτήν την ερευνητική μελέτη.**
   ☐ Διαφωνώ απόλυτα ☐ Διαφωνώ ☐ Ούτε συμφωνώ ούτε διαφωνώ ☐ Συμφωνώ ☐ Συμφωνώ απόλυτα
2. **Θα ένιωθα άγχος σχετικά με τη λήψη απόφασης για τη συμμετοχή του παιδιού μου στην ερευνητική μελέτη.**
   ☐ Διαφωνώ απόλυτα ☐ Διαφωνώ ☐ Ούτε συμφωνώ ούτε διαφωνώ ☐ Συμφωνώ ☐ Συμφωνώ απόλυτα
3. **Θα ένιωθα ενθουσιασμό για την ευκαιρία να συμμετάσχει το παιδί μου σε ερευνητική μελέτη.**
   ☐ Διαφωνώ απόλυτα ☐ Διαφωνώ ☐ Ούτε συμφωνώ ούτε διαφωνώ ☐ Συμφωνώ ☐ Συμφωνώ απόλυτα
4. **Θα φοβόμουν τους πιθανούς κινδύνους από τη συμμετοχή του παιδιού μου στην ερευνητική μελέτη.**
   ☐ Διαφωνώ απόλυτα ☐ Διαφωνώ ☐ Ούτε συμφωνώ ούτε διαφωνώ ☐ Συμφωνώ ☐ Συμφωνώ απόλυτα
5. **Θα ένιωθα υπερβολικό συναισθηματικό βάρος για να πάρω την απόφαση συμμετοχής.**
   ☐ Διαφωνώ απόλυτα ☐ Διαφωνώ ☐ Ούτε συμφωνώ ούτε διαφωνώ ☐ Συμφωνώ ☐ Συμφωνώ απόλυτα
6. **Θα ένιωθα πίεση να πάρω άμεσα απόφαση σχετικά με τη συμμετοχή του παιδιού μου.**
   ☐ Διαφωνώ απόλυτα ☐ Διαφωνώ ☐ Ούτε συμφωνώ ούτε διαφωνώ ☐ Συμφωνώ ☐ Συμφωνώ απόλυτα
7. **Φοβάμαι να δώσω συγκατάθεση, αλλά νιώθω τύψεις να αρνηθώ και να στερήσω από το παιδί μου πιθανά οφέλη από τη μελέτη.**
   ☐ Διαφωνώ απόλυτα ☐ Διαφωνώ ☐ Ούτε συμφωνώ ούτε διαφωνώ ☐ Συμφωνώ ☐ Συμφωνώ απόλυτα
8. **Θα ένιωθα εκνευρισμό αν μου ζητούσαν τώρα να συμμετάσχει το παιδί μου σε ερευνητική μελέτη.**
   ☐ Διαφωνώ απόλυτα ☐ Διαφωνώ ☐ Ούτε συμφωνώ ούτε διαφωνώ ☐ Συμφωνώ ☐ Συμφωνώ απόλυτα
9. **Θα νιώθατε άνετα να αρνηθείτε χωρίς τον κίνδυνο να επηρεαστεί η φροντίδα του παιδιού σας;**
   ☐ Ναι ☐ Όχι ☐ Δεν είμαι σίγουρος/η

QUESTIONNAIRE

Section A: Demographic Characteristics

1. Gender:
☐ Male ☐ Female

2. Age:
☐ <25 ☐ 25–34 ☐ 35–44 ☐ ≥45

3. Nationality/Origin:
Greek ☐ Other ☐ Please specify: ……………………………………..

4. Religion:
Christian (Orthodox/Catholic/Protestant) ☐ Muslim ☐ Atheist ☐ Other ☐

5. Educational level:
☐ Compulsory education ☐ Secondary education ☐ Higher/University education
☐ Postgraduate+

6. Occupation related to the healthcare/research sector:
Yes ☐ No ☐

If the answer is "Yes", please specify:
(e.g., nurse, physician, pharmacist, etc.) ……………………………………..

7. Relationship to the newborn:
☐ Mother ☐ Father ☐ Other

8. Do you have other children?
☐ Yes ☐ No

Section B: Information – Understanding of Clinical Research

1. Have you previously heard about clinical studies involving newborns?
☐ Yes ☐ No

2. How would you describe your level of knowledge about clinical research studies?
☐ None at all ☐ Limited ☐ Moderate ☐ Good ☐ Very good

3. In your opinion, what is the main difference between medical treatment and a clinical research study?
☐ Research is investigative – it does not provide direct benefits
☐ Research is an experimental treatment
☐ I do not know

4. Do you believe that clinical research studies are necessary for improving medical treatments?

☐ Strongly disagree ☐ Disagree ☐ Neither agree nor disagree ☐ Agree ☐ Strongly agree

5. Do you believe that the type of research study influences your decision to provide consent for your child's participation?

a) If the study involved a therapeutic intervention (e.g., administration of a medication or implementation of a new treatment approach):

How likely would you be to provide your consent for your child's participation?

☐ Not at all likely ☐ Slightly likely ☐ Fairly likely ☐ Very likely

b) If the study were observational and involved only the collection of data (with no intervention affecting your child):

How likely would you be to provide your consent for your child's participation?

☐ Not at all likely ☐ Slightly likely ☐ Fairly likely ☐ Very likely

6. Which form of information would be most helpful for you?
☐ Written information ☐ Verbal information ☐ Video ☐ Diagram ☐ It does not matter

Section C: Informed Consent Process

1. **Have you ever been asked to provide consent for your child's participation in a research study?**
   ☐ Yes ☐ No

   If you answered "Yes", please proceed to the following questions. If you answered "No", please proceed directly to Section D.

2. How well did you understand the informed consent form?
☐ Not at all ☐ Slightly ☐ Moderately ☐ Very well ☐ Completely

3. The amount of time you were given to make a decision was:
☐ Insufficient ☐ Sufficient ☐ Fully satisfactory

4. Did you feel comfortable refusing without the risk of affecting the care provided to your child?
☐ Yes ☐ No ☐ I am not sure

5. Were you given the opportunity to ask questions?
☐ No ☐ To some extent ☐ Yes, fully

6. Which emotions predominated when you were asked to provide consent?
(Select up to 2)

☐ Anxiety ☐ Fear ☐ Trust ☐ Hope ☐ Distrust ☐ Neutrality

<u>Section D: Trust and Relationship with Healthcare Professionals</u>

1. How much do you trust the medical staff of the Neonatal Intensive Care Unit (NICU)?
☐ Not at all ☐ Slightly ☐ Moderately ☐ Very much ☐ Completely

2. Do you feel that healthcare professionals respect your views and opinions?
☐ Yes ☐ No ☐ Partly

3. How well informed did you feel about your child's condition and about whether and how the study was related to your child's health?
☐ Not at all ☐ Slightly ☐ Moderately ☐ Completely

4. From whom would you prefer to receive information about research-related issues?
☐ Physician ☐ Nurse ☐ Psychologist ☐ All of them ☐ It does not matter to me

<u>Section E: Emotions Related to Participation in a Research Study</u>

1. I would feel anxious if I were approached to participate in this research study.
☐ Strongly disagree ☐ Disagree ☐ Neither agree nor disagree ☐ Agree ☐ Strongly agree

2. I would feel anxious about deciding whether my child should participate in the research study.
☐ Strongly disagree ☐ Disagree ☐ Neither agree nor disagree ☐ Agree ☐ Strongly agree

3. I would feel enthusiastic about the opportunity for my child to participate in a research study.
☐ Strongly disagree ☐ Disagree ☐ Neither agree nor disagree ☐ Agree ☐ Strongly agree

4. I would be afraid of the potential risks associated with my child's participation in the research study.
☐ Strongly disagree ☐ Disagree ☐ Neither agree nor disagree ☐ Agree ☐ Strongly agree

5. I would feel an excessive emotional burden in making the decision to participate.
☐ Strongly disagree ☐ Disagree ☐ Neither agree nor disagree ☐ Agree ☐ Strongly agree

6. I would feel pressure to make an immediate decision regarding my child's participation.
☐ Strongly disagree ☐ Disagree ☐ Neither agree nor disagree ☐ Agree ☐ Strongly agree

7. I am afraid to give consent, but I would feel guilty about refusing and potentially depriving my child of possible benefits from the study.
☐ Strongly disagree ☐ Disagree ☐ Neither agree nor disagree ☐ Agree ☐ Strongly agree

8. I would feel annoyed if I were asked at the moment for my child to participate in a research study.
☐ Strongly disagree ☐ Disagree ☐ Neither agree nor disagree ☐ Agree ☐ Strongly agree

9. Would you feel comfortable declining without the risk of your child's care being affected?
☐ Yes ☐ No ☐ I am not sure
